# A systematic review of the impact of type 2 diabetes on brain cortical thickness

**DOI:** 10.3389/frdem.2024.1418037

**Published:** 2024-06-13

**Authors:** Mahboubeh Motaghi, Olivier Potvin, Simon Duchesne

**Affiliations:** ^1^Faculté de Médecine, Université Laval, Québec City, QC, Canada; ^2^MEDICS Laboratory, Institut Universitaire de Cardiologie et de Pneumologie de Québec (IUCPQ), Québec City, QC, Canada; ^3^Département de Radiologie et Médecine Nucléaire, Université Laval, Québec City, QC, Canada

**Keywords:** type 2 diabetes (T2D), brain morphometry, cerebral cortical thickness, cortical brain atrophy, cognitive decline, microvascular disease

## Abstract

**Introduction:**

Type 2 diabetes (T2D) has been linked to cognitive impairment and dementia, but its impact on brain cortical structures in individuals prior to or without cognitive impairment remains unclear.

**Methods:**

We conducted a systematic review of 2,331 entries investigating cerebral cortical thickness changes in T2D individuals without cognitive impairment, 55 of which met our inclusion criteria.

**Results:**

Most studies (45/55) reported cortical brain atrophy and reduced thickness in the anterior cingulate, temporal, and frontal lobes between T2D and otherwise cognitively healthy controls. However, the balance of studies (10/55) reported no significant differences in either cortical or total brain volumes. A few reports also noticed changes in the occipital cortex and its gyri. As part of the reports, less than half of studies (18/55) described a correlation between T2D and hippocampal atrophy. Variability in sample characteristics, imaging methods, and software could affect findings on T2D and cortical atrophy.

**Discussion:**

In conclusion, T2D appears linked to reduced cortical thickness, possibly impacting cognition and dementia risk. Microvascular disease and inflammation in T2D may also contribute to this risk. Further research is needed to understand the underlying mechanisms and brain health implications.

## 1 Introduction

Recent epidemiological studies have identified several risk factors for dementia, including treatable health conditions such as hypertension, obesity, and diabetes (Livingston et al., 2020; Antal et al., 2022). While the exact causal links between these risk factors and dementia remain to be elucidated (Armstrong, 2013; Antal et al., 2022), multi-factorial, lifestyle-oriented interventions have shown promise at changing the trajectory of cognitive decline (Kivipelto et al., 2013), which underline the need to study these relationships further.

Diabetes, in particular, is a widespread epidemic that affects more than 425 million people worldwide (Adams et al., 2021) with type 2 diabetes (T2D) accounting for 90% of diabetes cases globally (Huang et al., 2022). Both type 1 and type 2 diabetes result in adverse effects not only for the pancreas but also the liver, kidneys, muscle, fat cells, and, as is now increasingly understood, the brain (Stephen Brunton, 2016). When compared to otherwise healthy controls, T2D patients showed a deficit in performance on cognitive tasks, especially memory (Moheet et al., 2015; Albai et al., 2019; Li et al., 2020a; Antal et al., 2022), and were found likely to develop cognitive problems, ranging from mild cognitive impairment (MCI) to dementia, between 10.8% and 17.5% (Li et al., 2018), with a risk ranging from 1.5- to 2.5-fold among the elderly (Ninomiya, 2014; Albai et al., 2019). T2D is therefore a key intervention target for dementia, as it is an altogether avoidable or otherwise treatable condition.

However, to understand its possible causal, catalytic, or other role in dementia, a proper understanding of T2D-related neurodegeneration in populations relatively free of dementia pathology is necessary. Indeed, while there are abnormalities associated with T2D that have been reported on brain magnetic resonance imaging (MRI), it remains unclear from the literature if general or specific cortical regions are more impacted by, or susceptible to, the presence of T2D throughout cognitively healthy aging (B van Harten et al., 2006). This understanding is necessary as such spatio-temporal relationships are logically necessary to link T2D with neurodegeneration and cognitive decline.

When investigating this issue, we found a growing body of evidence which suggests that T2D adversely affects brain anatomy in different areas (e.g., volumes of temporal, frontal, and limbic lobes; the hippocampus; cortical and subcortical gray matter), while other authors reported no association between T2D and brain morphology.

Thus, to clarify the likely impact of T2D on brain morphometry, we reviewed the literature on cerebral cortical thickness changes occurring in individuals with T2D without cognitive impairment.

## 2 Materials and methods

This review was conducted using the web-based software platform Covidence (*Covidence*) [Covidence, (n.d.)], per the recommendation in the PRISMA statement I (Moher et al., 2009).

### 2.1 Search strategy

We performed a systematic search of the literature of peer-reviewed journal articles published in English in four electronic bibliographic databases (PubMed, Embase, Ovid, and Web of Science), up to December 2023, with keywords related to cerebral cortical thickness (e.g., “cerebral cortex”, brain cortex”, “cerebral cortical”, “cortex volume”, “brain cortical volume”) and T2D (e.g., “diabetes mellitus, type 2”, “diabetes mellitus”, “type 2 diabetes mellitus”, “non-insulin-dependent diabetes mellitus”). In addition, we performed a manual search of references mentioned in articles found with our keyword search. Supplementary Table 1 depicts the strategies for the four databases.

### 2.2 Inclusion and exclusion criteria

Our selection criteria were that study participants needed to (a) have ascertained T2D; (b) be in selected age groups, that is individuals in either their midlife (45–65 years) and later-life (older than 65 years), based on the definitions used in the 2020 report of the Lancet Commission (Gill Livingston et al., 2020); (c) be cognitively healthy, i.e. free of objective impairment or dementia; and (d) be compared to a non-T2D and otherwise healthy control group. The presence of any other neurological or psychiatric disease or co-morbidity alongside T2D would also lead to excluding the paper.

### 2.3 Study selection

**Figure 1:**
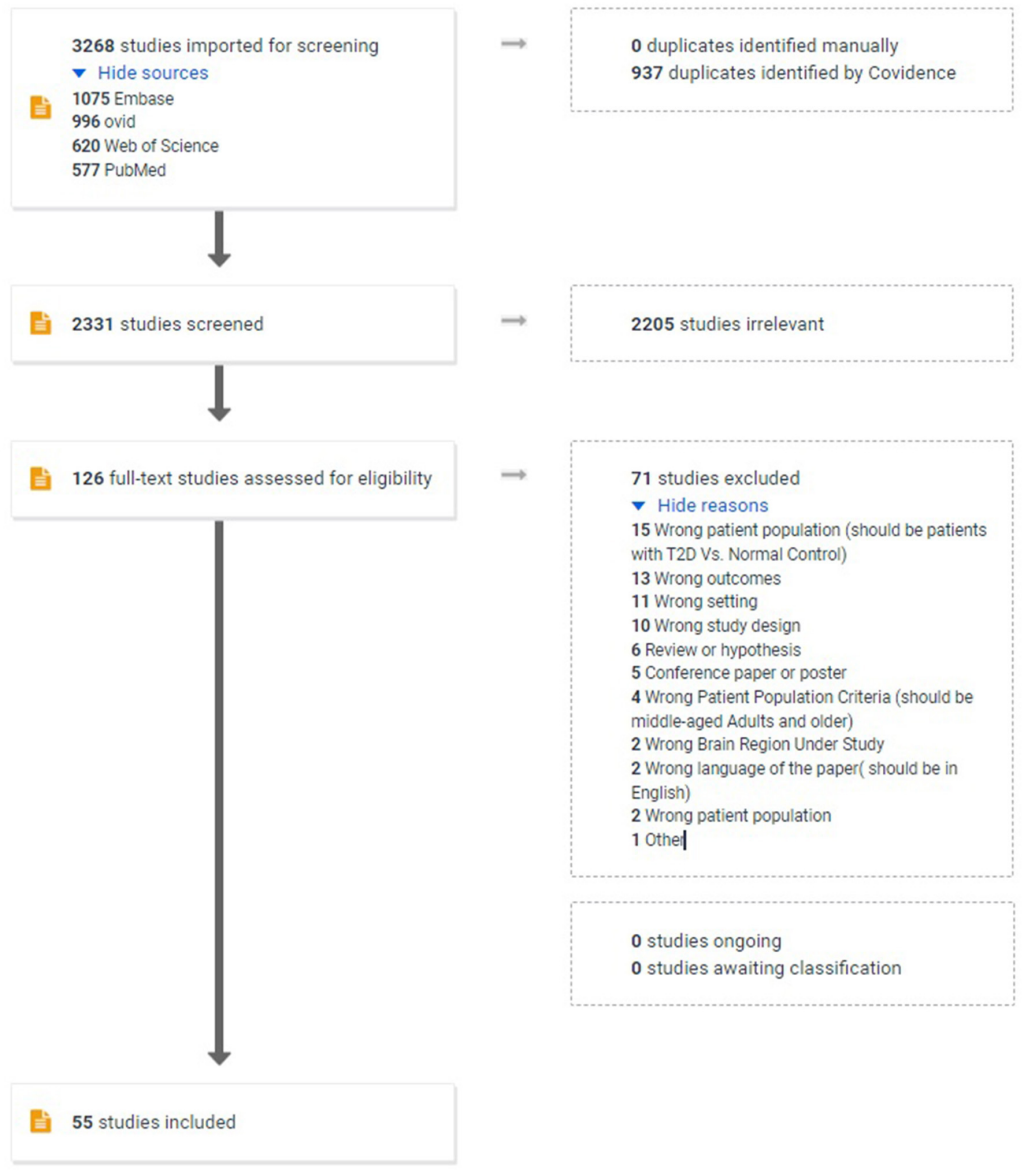
PRISMA Flow chart of the study selection procedure.

Our search identified 3,268 papers, among which 937 were recognized as duplicates and removed. Of the 2,331 unique studies, 126 independent studies were considered relevant after Title/Abstract screening by two independent reviewers (MM, SD). We then carried out a full-text review and excluded a further 69 papers based on eligibility criteria (Conference paper or poster; Review or Hypothesis paper; Wrong language; Wrong patient population or Population Criteria; Wrong setting; Wrong study design; Wrong Brain Region Under Study; Wrong outcomes). In fine, after a comprehensive review of 126 full-text papers, 55 studies were selected for inclusion and data extraction. Moreover, no additional paper was included after hand-searching the lists of references. Figure 1 depicts the PRISMA flow chart of our study selection process.

### 2.4 Data extraction

Data extraction was done by two reviewers (MM, SD) independently using a custom-designed data extraction electronic form in the Covidence software environment [Covidence, (n.d.)]. All disagreements were solved by consensus. Data consisted of:

1. General information: title, study ID, first author, country in which the study was conducted, year.
2. Study characteristics: aim of the study, study design, study funding sources, ethnicity of samples.
3. Participant cohort characteristics: total number of participants, T2D sample (size, age in years, % women, education, age at onset, disease duration), control sample (size, age in years, % women, education).
4. Imaging information: MRI field strength, manufacturers, T1-weighted MRI resolution, cortical thickness extraction software.
5. Results obtained: general results, statistical results, longitudinal results, longitudinal statistical results, cognitive changes (T2D vs. controls).

### 2.5 Quality assessment

Study quality assessment was carried out by two reviewers (MM, SD) independently, using the quality assessment tool for observational cohort and cross-sectional studies from the National Institute on Health, available at https://www.nhlbi.nih.gov/health-topics/study-quality-assessment-tools (*cf* . Supplementary Table 2). Studies were assessed for several aspects, based on their sample size and sample size justification, the aim of the study, the definition of independent and dependent variables, losses to follow up, eligibility criteria, the frequency of assessing exposures, and outcomes.

## 3 Results

### 3.1 Study characteristics

The 55 studies (see final references in Supplementary Table 1) included in the systematic review were published between 2003 and 2023 and originated from the United States, Canada, China, Australia, The Netherlands, and other countries including Portugal, Germany, Austria, Sweden, Poland, France, UK, Italy, and Spain. Out of 55 studies, 51 reported their sources of funding which are presented in detail as “Supplementary document: Sources of funding of the studies.”

Study design was mainly cross-sectional and retrospective (50), but for two with a longitudinal design, one with a prospective design and two with both cross-sectional and longitudinal results. The sample size of these studies varied from 20 to 4,053 participants; 70% of total participants were otherwise healthy controls (10,081 in total), while 30% of participants were individuals with T2D (4,264 participants altogether).

### 3.2 Study cohorts characteristics

Average age among study cohorts was distributed between 45.8 ± 7.6 years to 83.3 ± 2.6 years (Cherie M Falvey et al., 2013) for the control groups, and 47.7 ± 7.8 years (Shi et al., 2019; Chen et al., 2022) to 83.3 ± 3.1 years (C. M. Falvey et al., 2013) for T2D cohorts. The percentage of women ranged from 25% to 80% (Peng et al., 2014). Two thirds of studies (31 out of 55) reported the level of education for their participants, with controls and T2D both averaging 11.8 years of education. Across studies, the T2D patient group had a mean disease duration of 9.2 years, ranging from 2.0 ± 1.6 (Lee et al., 2021) to 20.1 ± 9.1 years, with a mean age of T2D onset across studies at 48.3 years. Approximately a third (15/55) of studies mentioned sample racial/ethnic diversity.

### 3.3 Study imaging characteristics

All studies acquired T1-weighted MRIs from several manufacturers at either 1.0, 1.5 or 3 Tesla scanner strength. Information regarding image resolution, repetition time, echo time, scan time, matrix size, field of view (FOV), slice thickness, and voxel size, alongside magnet strength, is presented in Supplementary Table 4. All studies used automated segmentation software to extract cortical thickness. Half of the studies (27 out of 55) used FreeSurfer (https://surfer.nmr.mgh.harvard.edu/); the rest used a variety of tools, including Statistical Parametric Mapping 8 software (https://fil.ion.ucl.ac.uk/spm/software/spm8/), Statistical Parametric Mapping 12 software (https://www.fil.ion.ucl.ac.uk/spm/software/spm12/). Multimodal Image Data Analysis System (MIDAS) (https://www.aps.anl.gov/Science/Scientific-Software/MIDAS), or custom MATLAB toolboxes.

### 3.4 General results

The main goal of each reviewed study was to assess the regional effects of T2D, whether in the presence or absence of other conditions, on cerebral cortical thickness as measured on T1-weighted MRI. Some studies dug deeper by, for example, evaluating the association between cognitive function and brain morphology in T2D patients, including the effect of various confounders such as age, years of education, and disease duration (Peng et al., 2014; Reynolds et al., 2023); investigating the relationship between fasting and non-fasting physiology with gray matter and white matter volume (Markus et al., 2017; Honea et al., 2022); measuring the association between neuropsychological deficits with structural and functional brain alternations (Garcia-Casares et al., 2014; Zhang et al., 2022, 2023); or comparing the effect of metabolic and vascular factors on cortical and subcortical gray matter structural alterations (Korf et al., 2006; Brundel et al., 2010; Tchistiakova et al., 2014; Bernardes et al., 2018; Cui et al., 2023; Moreno et al., 2023; Reynolds et al., 2023). Other studies compared the impact of T2D on brain morphology not only against controls, but also prediabetes (Jing et al., 2023; Monereo-Sánchez et al., 2023), major depression (Ajilore et al., 2010) or mild cognitive impairment and Alzheimer's disease (Moran et al., 2019; Palix et al., 2022).

**Figure 2:**
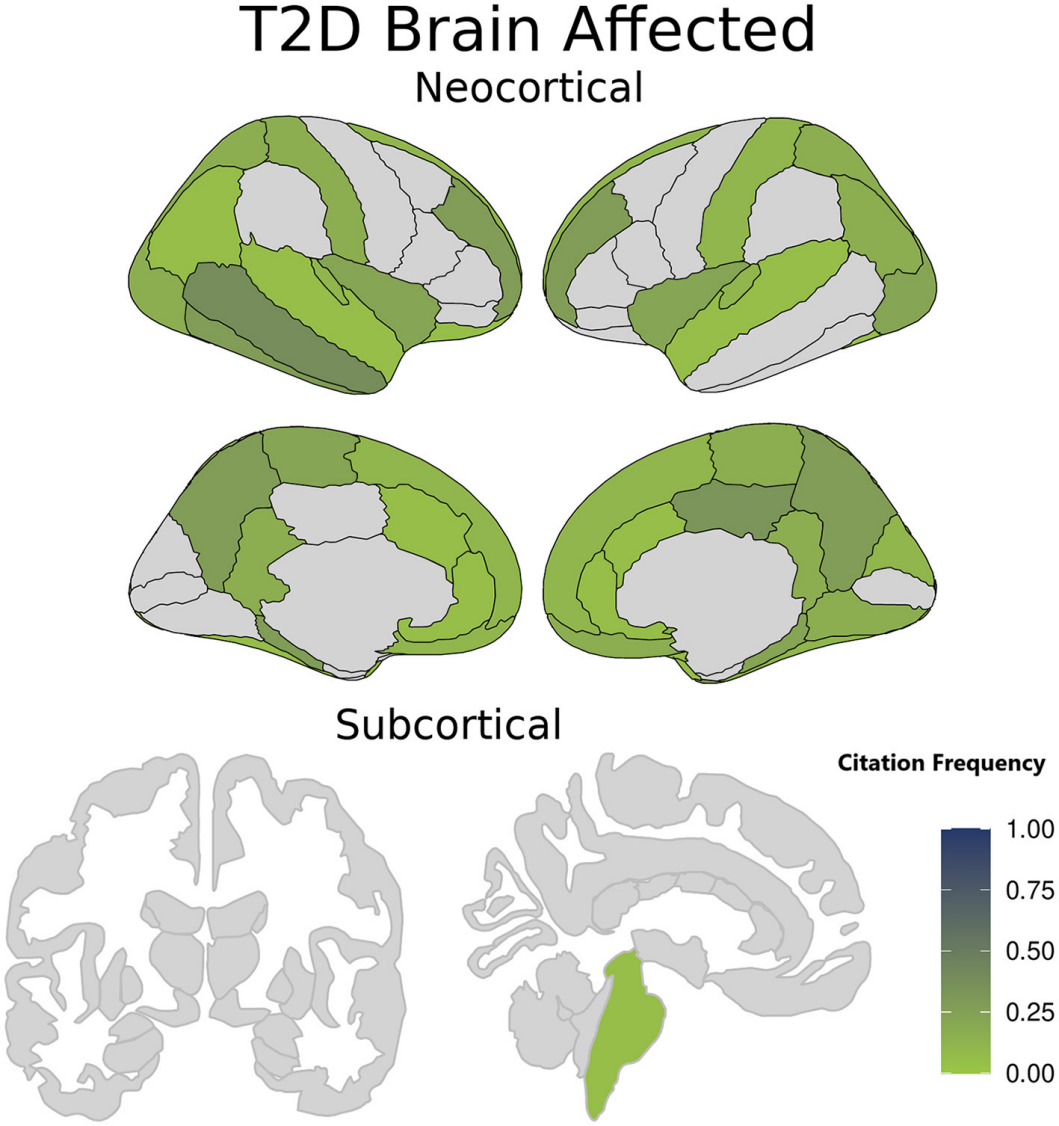
Brain structures affected in type 2 diabetes (T2D) patients compared with healthy controls. The color intensity in each region is related to the citation frequency, with darker shades suggesting higher mentions in the literature of a statistically significant T2D impact when compared to otherwise healthy controls. Darker colors signify brain regions more frequently cited as affected in T2D patients, while lighter Colors indicate brain regions less reported or cited in T2D studies. The color intensity is scaled from 0 to 1. A value of 0 represents brain regions not cited in any study, while a value of 1 signifies a region affected in all studies on T2D.

As detailed above, we extracted only those results pertaining to comparisons of cerebral cortical thickness between T2D and control cohorts. Because many studies also included subcortical volumes, these were also extracted even though they did not form part of our search or inclusion criteria. All are presented in Supplementary Table 4. Most papers (45 out of 55) have concluded that T2D is associated with cortical brain atrophy and a reduction in cortical thickness in several areas of the brain. Common to these studies are the left anterior cingulate; temporal lobe (including limbic areas); and frontal lobes. In addition to these areas, other brain regions are reported to be affected by T2D but with a lower occurrence across studies, including the left lateral occipital surface area, structural alterations in the occipital cortex, the left posterior cingulate gyrus, right isthmus cingulate gyrus, middle temporal gyrus, paracentral lobule, and transverse temporal gyrus, right superior frontal gyrus, left paracentral lobule, and left orbit of the renal cortex. Figure 2 demonstrates which brain structures seem affected in T2D patients when compared to otherwise healthy controls.

Finally, of the few discordant studies (10 out of 55) reporting no significant difference in cortical thickness between T2D and control groups, Wisse et al. (2014) further describe no association either between HbA1c and global brain volume; Moran et al. (2019), only the possibility of an indirect contribution with cognitive decline via its association with lower cortical thickness at baseline; in Liu et al. (2019), only region-based functional connectivity impairment in T2D patients compared with the control group; and in Zhang et al. (2023), T2D patients did not show cingulate gyrus thickness differences but displayed altered connectivity patterns, suggesting that functional issues may precede structural changes.

### 3.5 Impact of T2D-related factors and sex on cortical and subcortical changes

#### 3.5.1 Disease duration

Disease duration in T2D patients was statistically shown to be a factor related to cortical/sub-cortical decline in almost all studies. Specifically, Reynolds et al. (2023), found that longer duration of T2D is associated with reduced cortical thickness, gray matter volume, subcortical gray matter volume, and increased white matter hyperintensity volume. Only one discordant report concluded that T2D duration was not correlated with hippocampal volume (Li M. et al., 2020).

#### 3.5.2 HbA1C

Regarding HbA1C, indicative of diabetes control over the past 2–3 months, four studies did not report an association or interaction with T2D and brain matter (Hayashi et al., 2011; Cui et al., 2014; Wisse et al., 2014; Chen et al., 2017), while four studies found an association (Gold et al., 2007; Last et al., 2007; Zhang et al., 2015; Li et al., 2023). Specifically, in Li et al., an increase in visit-to-visit variability in HbA1c levels was linked to a higher risk of developing dementia and reduced hippocampal volume, emphasizing the significance of monitoring HbA1c levels for cognitive health in middle-aged and older adults without diabetes (Li et al., 2023).

#### 3.5.3 Sex

Few papers explicitly tested for differences in the effect of T2D on brain atrophy between men and women (Ajilore et al., 2010; Gabriel Bernardes et al., 2018; Moran et al., 2019). For example, Bernardes et al. (2018) reported that higher glucose levels and female sex were linked to a reduction in the left lateral occipital surface area, alongside a decrease in the left paracentral area. After adjusting for group allocation, only the association between being female and lower left lateral occipital and paracentral surface areas remained statistically significant (Bernardes et al., 2018). Despite contradictory findings regarding the impact of T2D on brain atrophy changes in men and women, three studies have shed light on the influence of sex differences on brain structure and cognitive function in individuals with and without T2D (Manschot et al., 2006; van Velsen et al., 2013). The first study did not find a significant association between sex and cognition but revealed that men with T2D may experience more pronounced subcortical atrophy (Manschot et al., 2006). The second study demonstrated that women generally have a thicker cortex, potentially influenced by variations in estrogen levels, while men may exhibit a greater decline in cortical thickness, likely due to their relatively higher cardiovascular risk profiles (van Velsen et al., 2013) Thirdly, Bernardes et al. (2018) found that cortical alterations were primarily influenced by sex, while the subcortical alterations were associated with factors such as BMI, total cholesterol, and age.

### 3.6 Methodological considerations

There does not seem to be any variability between studies reporting an association and that used Freesurfer (nearly half, or 20/45) compared to those that used other tools for cortical thickness segmentation. Moreover, six of the ten discordant studies also used Freesurfer (version 5.3.0 or later), which would tend to indicate that there is no technique bias in this result. Other sources of variability that were not tested include field strength and imaging protocols (resolution, repetition and echo time, matrix size and slice thickness, and field of view), which may affect the accuracy of cortical thickness segmentation regardless of the tool used.

## 4 Discussion

### 4.1 General association between T2D and brain cortical thickness

This systematic review examined the regional effect of T2D on brain cortical thickness by reviewing peer-reviewed articles published in the past 23 years. In our review, 45 out of 55 studies supported in general an association between T2D and cerebral cortical brain atrophy. More importantly, this reduction in cortical thickness did not appear to be distributed equally. This increased atrophy may, in part, underlie greater rates of decline in cognition in individuals with high blood glucose levels (Shaw et al., 2017), higher glycated hemoglobin (HbA1c) (Li et al., 2023), and higher fasting glucose (Honea et al., 2022).

Generally, the reviewed studies indicate that there is a negative association between T2D and brain structural measures, including gray matter volume, cerebral cortex thickness, and for those report which included measurements, subcortical volumes. The brain areas affected by these changes show both a reduction in volume and a decrease in thickness. As shown in Figure 2, these changes are particularly noticeable in the left anterior and posterior cingulate, superior, frontal, and middle temporal lobes, the latter encompassing limbic regions.

Besides these, other brain areas have also been reported to be affected by T2D but with reduced frequency, including the left lateral occipital cortex, left posterior and right isthmus of the cingulate gyrus, middle temporal gyrus, paracentral lobule, transverse temporal gyrus, right superior frontal gyrus, left paracentral lobule, and left orbit of the renal cortex (see Supplementary Table 4). Since these findings were not often replicated, it is possible that they came about as spurious expressions of imaging noise or study group bias (see Section T2d characteristics and brain cortical thickness).

### 4.2 T2D characteristics and brain cortical thickness

The relationship between elevated glucose and/or insulin levels and decreased gray matter volume is not yet well understood. However, one potential explanation for this association is the disruption of insulin signaling (Markus et al., 2017; Antal et al., 2022; Honea et al., 2022). The brain heavily relies on continuous blood flow, and conditions such as hyperglycemia and hyperinsulinemia can result in microvascular changes, endothelial dysfunction, and atherosclerosis, which in turn may impact cerebral protein synthesis. High glucose levels can also harm neurons, causing oxidative stress, mitochondrial dysfunction, cellular energy deficits, and neuronal dysfunction and death. Insulin plays a crucial role in neuronal survival, protein synthesis, and synaptic metabolism, and chronic hyperinsulinemia can cause insulin receptors in the blood-brain barrier to become deregulated, leading to brain insulin resistance and neural aging and degeneration (Markus et al., 2017; Zhang et al., 2023). The disruption of insulin signaling can result in impaired metabolism, synaptic plasticity, microtubule damage, and genetic activation during cell death (Markus et al., 2017). We therefore expected that cortical thickness changes would be explained by the principal characteristics of diabetes (e.g., disease duration, HbA1C, insulin resistance, fasting glucose).

However, there is uncertainty around the effect of these variables on cortical brain atrophy, given that while many studies reported an association between brain volume measurements and diabetes characteristics such as diabetes duration, HbA1c, and hypoglycemic episodes (Korf et al., 2006; Manschot et al., 2006; van Harten et al., 2006; de Bresser et al., 2010; Garcia-Casares et al., 2014; Ninomiya, 2014; Wisse et al., 2014; Moheet et al., 2015; Peng et al., 2015; Brunton, 2016; Buss et al., 2018; Li et al., 2018, 2023; Li M. et al., 2020; Livingston et al., 2020; Roy et al., 2020; Honea et al., 2022), the specific factors associated with these changes varied across studies, and not all studies found statistically significant associations between all factors and all brain volume measurements. As a result, further research is needed to clarify the relationship between T2D-related variables and cortical brain atrophy. Other factors commonly related to T2D, such as BMI, hypertension, or dyslipidemia, did not seem to independently contribute to hippocampal volume loss in the studies reporting on this structure, but rather their effects were subsumed by HbA1c.

It should be also mentioned that although the impact of sex on the effect of T2D on brain cortical atrophy was expected to be a determinative factor, among the studies reviewed many of them did not directly address the role of sex in the relationship between T2D and brain cortex atrophy. Instead, they put their focuses on the overall differences between patients with T2D and controls in terms of brain volume and cortical atrophy. It is worth noting that many studies on this topic have included relatively small sample sizes (*cf* . Supplementary Table 3), which can limit the ability to detect sex differences.

### 4.3 T2D characteristics and brain subcortical thickness

Based on the reviewed studies which encompassed a total of 17 papers reporting subcortical volumes, the impact of T2D on subcortical regions remains inconclusive. Some of the studies revealed significant hippocampus atrophy in T2D compared with healthy controls (den Heijer et al., 2003; Korf et al., 2006; Manschot et al., 2006; Gold et al., 2007; Last et al., 2007; Bruehl et al., 2009; Brundel et al., 2010; Hayashi et al., 2011; Falvey et al., 2013; Peng et al., 2015; Zhang et al., 2015; Bernardes et al., 2018; Li M. et al., 2020; Lee et al., 2021; Honea et al., 2022; Monereo-Sánchez et al., 2023; Moreno et al., 2023). The effect sizes for hippocampal atrophy ranged from a minimum of 0.07 to a maximum of 1.365, suggesting a moderate to large effect of T2D on hippocampal volume reduction, with considerable variability across studies. Some of the studies found subcortical atrophy in regions other than the hippocampus, including the amygdala, with effect sizes ranging from 0.57 to 2.31 (den Heijer et al., 2003; Gold et al., 2007; Bernardes et al., 2018; Roy et al., 2020), CA1 and subiculum subfields (Zhang et al., 2015; Monereo-Sánchez et al., 2023), thalamus (Bernardes et al., 2018; Bhaswati Roy et al., 2020; Dong et al., 2022; Jing et al., 2023), nucleus accumbens (Chang Li et al., 2020a), insula, cingulate gyrus, caudate and cerebellum.

Some studies have indicated a potential direct contribution of T2D to the development of Alzheimer's neuropathology, as evidenced by atrophy in the hippocampus and amygdala, independent of other factors such as atherosclerosis or cerebrovascular disease (den Heijer et al., 2003; Gold et al., 2007). It has been suggested that T2D primarily affects subcortical areas and potentially leading to more significant cognitive impairments associated with subcortical structure reductions (Bruehl et al., 2009; Li et al., 2020b; Lee et al., 2021). The association between T2D and hippocampal volume loss is thought to be related to factors like hyperglycemia and advanced glycation end-products. However, not all studies have found significant associations between T2D and atrophy in subcortical regions, suggesting that the link between T2D and dementia may be more related to reductions in brain reserve rather than a direct link to Alzheimer's disease (Bruehl et al., 2009; Li et al., 2020b). It is proposed that hippocampal-based declarative memory is one of the cognitive domains affected early in T2D, with additional impairments emerging as the disease progresses (Bruehl et al., 2009; Li et al., 2020b). While some studies reported no significant associations (Schmidt et al., 2004; van Velsen et al., 2013; Wisse et al., 2014; Moran et al., 2015; Wennberg et al., 2016; Chen et al., 2017; Coutinho et al., 2017; Buss et al., 2018; Li et al., 2018; Li M. et al., 2020), the presence of effect sizes within the reported range indicates a potential trend of subcortical structural alterations associated with T2D. However, due to the limited number of reports for the amygdala, thalamus, and caudate, further research specifically for these regions is needed to establish more definitive conclusions regarding the impact of T2D. Overall, it can be suggested that diabetes-associated memory impairments may be linked to hippocampal pathology, but not necessarily to other subcortical structures.

### 4.4 Impact of race/ethnicity

Based on the studies reviewed, there is evidence that the incidence of T2D varies by race and ethnicity. According to one study (Korf et al., 2006), the incidence of T2D was highest among American Indians/Alaska Natives, followed by African Americans, Hispanic Americans, Asian Americans, and then non-Hispanic Whites. According to another study (Manschot et al., 2006), Native Hawaiians/Pacific Islanders had the highest prevalence of T2D in their sample, followed by Hispanic/Latino Americans, African Americans, Native Americans/Alaska Natives, and Asian Americans. T2D prevalence was lowest among non-Hispanic Whites. Even though these studies were not designed to evaluate these effects, they support the idea that T2D disproportionately affects certain racial and ethnic groups, and that understanding these differences can help prevent and treat the disease more effectively. Further details can be found in Supplementary Table 3.

### 4.5 Source of discrepancies

Despite most studies demonstrating a relationship between T2D and brain cortical atrophy, a handful (10/55) concluded the opposite. It is worth noting that these discordant papers present differences in the sample size, population characteristics, imaging methods, and/or diabetes duration when compared to the remainder of studies, which can affect the results. These factors are discussed further below.

The MRI parameters used in the reviewed studies can have a significant impact on the quality of the images and therefore on the ability to detect changes in brain cortical thickness in individuals with T2D (van Harten et al., 2006). The strength of the MRI scanner, measured in Tesla (T), can affect the signal-to-noise ratio of the images, making it easier to detect subtle changes in cortical thickness. Higher field strength scanners (3T or above) tend to produce higher-resolution images than lower field strength scanners (1.5T or below) (van Harten et al., 2006). Interestingly, half of the discordant studies (5/10) used 1.5T scanners. However, some concordant studies also used the same scanner strength, indicating that strength alone may not be the determining factor in producing consistent results.

Differences may also arise due to there being multiple scanner manufacturers, i.e., different imaging protocols and settings, which can affect the quality of the images and the ability to detect changes in cortical thickness (George et al., 2020).

The use of different software programs will also produce varying cortical thickness measurements from otherwise identical MRI. We have already noted that Freesurfer was used in the majority of studies, a software that has been shown to have high accuracy and reliability in measuring cortical thickness (Gronenschild et al., 2012). Other toolkits included SPM8 (Garcia-Casares et al., 2014; Gao et al., 2023) and SPM12 (Roy et al., 2020; Palix et al., 2022; Zhang et al., 2022), MRIcroN (Roy et al., 2020), REST (Roy et al., 2020), VSRAD (Hayashi et al., 2011), MarsBaR (Ferreira et al., 2017), MEDx (Korf et al., 2006), PM12 (Crisóstomo et al., 2021) and CAT12 (Crisóstomo et al., 2021), Computational Anatomy Toolbox 12 (CAT12: http://www.neuro.uni-jena.de/cat/) (Chen et al., 2022; Zhang et al., 2023). We observed that almost all discrepant studies (6 out of 10) predominantly utilized newer versions of Freesurfer, specifically versions 5.3 and 6.0. However, given its use in concordant studies, it may not warrant further research on software version for these imaging outcomes.

### 4.6 Limitations

Limitations of the studies included in this review may hinder our ability to draw significant conclusions. One limitation is the potential for publication bias, as studies with statistically significant results are more likely to be published, leading to a potential overrepresentation of positive findings in the review. Another limitation is the restriction of the search to specific databases, which may result in the exclusion of relevant studies from other databases, thereby limiting the comprehensiveness of the review.

Furthermore, most studies included in the review were cross-sectional, lacking longitudinal data, which hinders the establishment of causal relationships and the assessment of long-term effects. The variations in sample sizes and sex distributions across studies can also affect the generalizability of the findings. The heterogeneity in study designs and outcomes prevented the conduct of a meta-analysis, which could have provided a more comprehensive synthesis of the results. Moreover, the exclusion of results from studies that examined T2D in conjunction with other comorbid conditions, such as obesity, hypertension, and depression, due to the specific objective of the systematic review, limits the generalizability of the findings. Additionally, the review focused exclusively on English-language papers, introducing a potential language bias, and excluding relevant information published in other languages. Despite these limitations, the review aimed to comprehensively assess the impact of T2D on brain cortical atrophy by synthesizing insights from the available literature. The findings provide meaningful observations and suggest potential implications for future research in this field.

## 5 Conclusion

In summary, our systematic review indicates that patients with T2D have reduced cortical thickness mainly in the left anterior cingulate, temporal lobe, frontal lobe, and limbic lobe, as well as cortical gray matter. From those papers that reported additional results, hippocampal and subcortical gray matter atrophy was also present. It seems that these regions have commonality with AD vulnerable areas, as suggested by Wennberg et al. (2016) and Palix et al. (2022). Such evidence could be another confirmation of the commonality of T2D vulnerable brain regions with that of AD. It is also possible that the changes in brain structure may be a result of microvascular disease or chronic inflammation, which are commonly seen in individuals with diabetes. Overall, the research suggests that T2D may have a negative impact on brain health, and further studies are needed to fully understand the mechanisms underlying these changes.

## Data availability statement

The original contributions presented in the study are included in the article/Supplementary material, further inquiries can be directed to the corresponding author.

## Author contributions

MM: Writing – review & editing, Writing – original draft, Visualization, Software, Formal analysis, Data curation. OP: Writing – review & editing, Visualization, Software. SD: Writing – review & editing, Validation, Supervision, Resources, Methodology, Investigation, Funding acquisition, Conceptualization.
