# Supplementary material for: A systematic review of the impact of type 2 diabetes on brain cortical thickness
Source: Front Dement. 2024 Jun 13;3:1418037. doi: 10.3389/frdem.2024.1418037 (PMC11285553; doi:10.3389/frdem.2024.1418037)
Supplement: Supplementary file 1 [file Table_1.docx]

| **Bibliographic Databases** | **Search Methods** | **Number of studies found** |
| --- | --- | --- |
| PubMed | (((((Cerebral Cortex/[MeSH Terms]) OR ("Cerebral Cortex"[Title/Abstract] OR "brain cortex"[Title/Abstract])) OR ("brain cortical volume"[Title/Abstract])) OR ("cerebral cortical volume"[Title/Abstract])) OR ("cortex volume"[Title/Abstract])) AND ((((((Diabetes Mellitus, Type 2/[MeSH Terms]) OR ("diabetes mellitus"[Title/Abstract])) OR ("diabetes type 2"[Title/Abstract])) OR (T2D[Title/Abstract])) OR ("Type 2 Diabetes Mellitus"[Title/Abstract])) OR ("non-insulin dependent diabetes mellitus"[Title/Abstract])) | 952 |
| Embase | ('cerebral cortex/'/exp/mj OR 'cerebral cortex':ti,ab,kw OR 'brain cortex':ti,ab,kw OR 'cerebral cortical volume':ti,ab,kw OR 'cortex volume':ti,ab,kw OR 'brain cortical volume' OR (('brain'/exp OR brain) AND cortical AND ('volume'/exp OR volume))) AND ('diabetes mellitus, type 2':ti,ab,kw OR 'diabetes mellitus':ti,ab,kw OR t2d:ti,ab,kw OR 'type 2 diabetes mellitus':ti,ab,kw OR 'non-insulin dependent diabetes mellitus':ti,ab,kw) AND [2000-2021]/py | 435 |
| Ovid | ("Diabetes Mellitus, Type 2" /) OR (("diabetes mellitus" or "diabetes type 2" or T2D or "Type 2 Diabetes Mellitus" or "non insulin dependent diabetes mellitus").ti,ab.) AND (("Cerebral Cortex"/) OR (("Cerebral Cortex" or "brain cortex").ti,ab.) OR (("brain cortical volume" or "cerebral cortical volume" or "cortex volume").ti,ab.)) | 295 |
| Web of Science | (((((TS= (Cerebral Cortex)) OR TS= (brain cortex)) OR TS= (brain cortical volume)) OR TS= (cerebral cortical volume)) OR TS= (cortex volume)) AND ((((((TS= (Diabetes Mellitus, Type 2)) OR TS= (diabetes mellitus)) OR TS= (diabetes type 2)) OR TS=(T2D)) OR TS= (Type 2 Diabetes Mellitus)) OR TS= (non-insulin dependent diabetes mellitus)) | 1083 |
| Final References | (Ajilore et al., 2010; Gabriel Bernardes et al., 2018; Hannah Bruehl et al., 2009; Manon Brundel et al., 2010; Stephanie S Buss et al., 2018; Chen et al., 2015; Zhiye Chen et al., 2017; Choi et al., 2020; Artur Martins Coutinho et al., 2017; Crisóstomo et al., 2021; Xingran Cui et al., 2014; Jeroen De Bresser et al., 2010; Tom den Heijer et al., 2003; Cherie M Falvey et al., 2013; Ferreira et al., 2017; Garcia-Casares et al., 2014; S. Gold et al., 2007; Kumi Hayashi et al., 2011; Esther SC Korf et al., 2006; Kumar et al., 2008; David Last et al., 2007; Chang Li et al., 2018; Chang Li et al., 2020; MengChun Li et al., 2020; Liu et al., 2019; Sanne M Manschot et al., 2006; M. Markus et al., 2017; Chris Moran et al., 2015; Moran et al., 2019; Bo Peng, Zhiye Chen, et al., 2015; Peng et al., 2014; Bhaswati Roy et al., 2020; Reinhold Schmidt et al., 2004; Shaw et al., 2017; Shi et al., 2019; Ekaterina Tchistiakova et al., 2014; Evert FS van Velsen et al., 2013; Alexandra MV Wennberg et al., 2016; Laura EM Wisse et al., 2014; Y.-W. Zhang et al., 2015) | 40 |

**Table 1.** Search strategy for T2D and cerebral cortical thickness change
