# Supplementary material for: A systematic review of the impact of type 2 diabetes on brain cortical thickness
Source: Front Dement. 2024 Jun 13;3:1418037. doi: 10.3389/frdem.2024.1418037 (PMC11285553; doi:10.3389/frdem.2024.1418037)
Supplement: Supplementary file 3 [file Table_3.DOCX]

| **#** | **Reference of Original publication** | **Year** | **Study_Design** | **#Patients_T2D** | **Age_T2D** | **%Of_ Women_T2D** | **Education_T2D** | **Disease_Duration** | **Age_at_Onset** | **# Healthy** | **Age** | **% Of Women** | **Education** |
| --- | --- | --- | --- | --- | --- | --- | --- | --- | --- | --- | --- | --- | --- |
|  |  |  |  |  |  |  |  |  |  |  |  |  |  |
| 1 | Schmidt et al., 2004 | 2004 | Cross sectional study | 114 | 69.3 ± 3.0 | 71(62.3%) | 8.5 ±4.7 | - | - | 1138 | 68.8 ±3.2 | 584 (51.3 %) | 9.0 ±4.2 |
| 2 | Coutinho et al., 2017 | 2017 | Cross sectional study | - | - | - | - | - | - | - | - | - | - |
| 3 | Buss et al., 2018 | 2018 | Cross sectional study | 28 | 66.3±1.5 | 13 (46%) | 15.5±0.5 | - | - | 27 | 61.7±1.6 | 12 (44%) | 15.8±0.6 |
| 4 | Markus et al., 2017 | 2017 | Cross sectional study | 64 | 64 (54, 69) | 26(40.6%) | Less than 10 years: 23.4% | - | - | 759 | 45 (36,55) | 474(62.5%) | Less than 10 years: 7.4 % |
|  |  |  |  |  |  |  | 10 years: 45.3% |  |  |  |  |  | 10 years: 57.3% |
|  |  |  |  |  |  |  | More than 10 years: 31.3% |  |  |  |  |  | More than 10 years: 35.3% |
| 5 | Garcia-Casares et al., 2014 | 2014 | Cross sectional study | 25 | 60.0±4.6 | 8(32%) | 18.3±3.6 | - | - | 25 | 57.8±5.4 | 11 (44%) | 18.9±4.0 |
| 6 | (Roy et al., 2020 | 2020 | Cross sectional study | 34 | 56.8±7.1 | 19(55.88%) | 15.4±2.1 | 10.5±7.7 | 46.3±10.47 | 88 | 54.4±5.1 | 42(47.73%) | 16.6±2.7 |
| 7 | Falvey et al., 2013 | 2013 | Cross sectional study | 85 | 83.3 ±3.1 | 39(45.9%) | - | - | - | 223 | 83.3±2.6 | 142 (62.8%) | - |
| 8 | (Peng et al., 2014 | 2014 | Cross sectional study | 10 | - | 8(80%) | - | - | - | 10 | - | 7 (70%) | - |
| 9 | Last et al., 2007 | 2007 | Cross sectional study | 26 | 61.6±6.6 | 13 (50%) | - | 12.9±11.3 | 48.7± 13.08 | 25 | 60.4± 8.6 | 12 (48%) | - |
| 10 | Bernardes et al., 2018 | 2018 | Case control study | 13+15 | 60.44 ± 5.05 (50.86-70.24) | 15/13 (53%) | - | 8.15± 4.81 (1_20) | - | 14+17 | 57.08± 7.10(41.03-66.92) | 16/15 (51.6) | - |
| 11 | Ferreira et al., 2017 | 2017 | Cross sectional study | 24 | 58.58 ± 8.63 | 11 (46%) | - | 7.96 ± 7.86 | 50.62± 11.67 | 27 | 59.89± 5.93 | 14 (52%) | - |
| 12 | Hayashi et al., 2011 | 2011 | Cross sectional study | 61 | 74±7 | 27 (44.3%) | 9±2 | 16±10 | 58±12.21 | 53 | 74±5 | 28 (52.8%) | - |
| 13 | Kumar et al., 2008 | 2008 | Cross sectional study | 26 | 57.85±8.53 | - | - | 118.15±98.94 (months) = (9.85 ± 8.245 years) | 48 ± 11.86 | 25 | 53.24±9.12 | - | - |
| 14 | Zhang et al., 2015 | 2015 | Cross sectional study | 80 | 57.49 (9.04) | 45 (56.25%) | 9.63 (3.94) | 7.00 (6.67) | 50.49(11.23) | 80 | 57.83 (10.31) | 51 (63.75%) | 10.08 (4.22) |
| 15 | Bruehl et al., 2009 | 2009 | Cross sectional study | 41 | 59.05±8.35 | 19(46.3%) | 14.96±2.36 | 7.00±6.40 | 52.05± 8.68 | 47 | 60.02±7.96 | 23(49%) | 15.81±1.92 |
| 16 | Gold et al., 2007 | 2007 | Cross sectional study | 23 | 59.2±8.4 | 12(52.2%) | 15.6±2.3 | 6.0± 6.3 | 53.2±10.5 | 23 | 59.9±8.6 | 12(52.2%) | 15.9±1.9 |
| 17 | Korf et al., 2006 | 2006 | Cross sectional study | 202 | 81±5.1 | 0 | 10.3±3.2 | - | - | 204 | 81.3±4.9 | - | 10.2±3.3 |
| 18 | Wisse et al., 2014 | 2014 | Cohort study | 235(120+61+54) | SMART-Medea: 64.0 ± 8.7 | SMART-Medea: 22 (18%) | - | SMART-Medea: 6 (1,16) | SMART-Medea: | 580 (502 + 30 +48) | SMART-Medea: 60.9 ± 9.7 | SMART-Medea: 95(19%) | - |
|  |  |  |  |  | UDES1: 70.2± 5.4 | UDES1: 31 (51%) |  | UDES1: 12 (7,22) | UDES1: |  | UDES1: 68.6± 4.4 | UDES1: 17(57%) |  |
|  |  |  |  |  | UDES2: 70.9 ± 3.9 | UDES2:18 (41%) |  | UDES2: 9 (3,22) | UDES2: |  | UDES2: 71.2± 4.8 | UDES2: 18(37%) |  |
|  |  |  |  |  |  |  |  |  |  |  |  |  |  |
|  |  |  |  |  | Total: 68.37± |  |  |  |  |  |  |  |  |
| 19 | Li et al., 2020 | 2020 | come from one cohort study and two case-control studies | 36 | 64.11± 8.22 | 17 (47.2%) | 11.14± 4.16 | 8.91± 6.25 | 55.2 ± 10.33 | 47 | 2.28 ± 8.66 | 23 (49%) | 1.79 ± 3.21 |
| 20 | Manschot et al., 2006 | 2006 | Cross sectional study | 113 | 66.1±5.6 | 57(50.4%) | 4(3-5) | 8.8±6.2 | 57.3±8.35 | 51 | 65.1±5.3 | 29 (57%) | 4 (3-5) |
| 21 | de Bresser et al., 2010 | 2010 | Cross sectional study | 55 | 65.9± 5.4 | 29 (53%) | 4.2± 1.5 | 9.5 ± 6.6 | 56.4 ± 8.53 | 28 | 64.2± 4.3 | 16 (57%) | 4.5 ± 1.3 |
| 22 | Ajilore et al., 2010 | 2010 | Cohort study | 26 | 81 (8.49) | 18 (69%) | 4.58 (4.04) | 9.85 (8.24) | 71.15 (11.83) | 20 | 15 (8.23) | 15 (75%) | 5.20 (2.67) |
| 23 | (Tchistiakova et al., 2014 | 2014 | Cross sectional study | 18 | 71.8± 5.6 | 8 (44.44%) | - | 10.9 ± 6.6 | 60.9 ± 8.65 | 22 | 73.4± 6.2 | 12 (54.54%) | - |
| 24 | Cui et al., 2014 | 2014 | Cross sectional study | 43 | 65.52± 8.73 | 23 (53.5%) | 15.17±3.62 | 13.33±6.81 | 52.19 ± 14.09 | 26 | 65.21±10.23 | 12 (46.2%) | 15.92±3.02 |
| 25 | Wennberg et al., 2016 | 2016 | Cohort study | - | - | - | - | - | - | - | - | - | - |
| 26 | Shaw et al., 2017 | 2016 | Cross sectional study | 48 | 62.9±1.3 | 22 (47%) | 13.6±2.6 | - | - | 212 | 63.1±1.5 | 116 (55%) | 14.3 ±2.5 |
| 27 | Li et al., 2018 | 2018 | Other: longitudinal design | 30 | 54.97±5.54 | 11(37%) | 11.90±2.92 | 7.93±5.98 | 47.04±8.15 | 30 | 53.17±6.57 | 16 (53.3%) | 11.80±2.99 |
| 29 | Shi et al., 2019 | 2019 | Cross sectional study | 60 | 51.4 ± 7.3 | 17 (28.3%) | 11.02 ±4.0 | 6.70±6.1 | 40.38±9.51 | 28 | 50.5±6.4 | 13(46.43%) | 10.86±3.9 |
| 30 | Moran et al., 2019 | 2019 | Cross sectional study | 124 | 75.5±6.2 | 39 (31%) | 15.1±3.5 | - | - | 693 | 75.1±6.9 | 303(44%) | 15.6±3.0 |
| 31 | Crisóstomo et al., 2021 | 2021 | cross-sectional and longitudinal | 86 | 60.76 ±7.89 | 35 (40.7%) | - | 12.05±8.88 | 47.73 ±10.26 | 40 | 57.73±8.06 | 20(50%) | - |
| 32 | den Heijer et al., 2003 | 2003 | Cross sectional study | 41 | 77±8 | 14(34%) | primary only: 38% | - | - | 465 | 73±8 | 232 (50%) | Primary Only: 30% |
| 33 | Brundel et al., 2010 | 2010 | Cross sectional study | 56 | 70.0±5.2 | 29 (51%) | - | 13.6±6.8 | 56.4±8.56 | 30 | 68.1±4.3 | 17 (57%) | - |
| 34 | van Velsen et al., 2013 | 2013 | Cross sectional study | 94 | 68.4±7.3 | 42 (44.68%) | - | - | - | 928 | 68.4±7.3 | 492 (53.02%) | - |
| 35 | Chen et al., 2015 | 2015 | Cohort study | 11 | 61.2 | 8(73%) | - | 13.9 | 47.3 | 11 | 56.2 | 8(73%) | - |
| 36 | Moran et al., 2015 | 2015 | cross-sectional and longitudinal | 124 | 75.5±6.2 | 39 (31.5%) | >=6 | - | - | 692 | 74.1±7.0 | 303 (44%) | >=6 |
| 37 | Peng et al., 2015 | 2015 | Cross sectional study | 18 | 61.2 | 11(61%) | - | - | - | 17 | 62.2 | 11(65%) | - |
| 38 | Chen et al., 2017 | 2017 | Cross sectional study | 64 | 61.2±7.8 | - | - | 13.2±5.6 | - | 16 | 59.6±6.1 | - | - |
| 39 | Liu et al., 2019 | 2019 | Cross sectional study | 94 | 49.11 ±9.68 | 20(61%) | 13.3 ±3.53 | 3.2 | 45.91 ±9.68 | 24 | 52.50 ±9.66 | 14(58.3%) | 11.88 ±3.30 |
| 40 | Choi et al., 2020 | 2020 | Cross sectional study | 114 | 57.1 (51.2-61.4) | 11(39%) | - | 10.2 ± 7.4 | 46.9 | 47 | 55.0 (52.3-60.3) | 29 (62%) | - |
| 41 | Dong et al., 2022 | 2022 | Cross sectional study | - | - | - | - | - | - | - | - | - | - |
| 42 | Honea et al., 2022 | 2021 | Other: longitudinal study | - | - | - | - | - | - | - | - | - | - |
| 43 | Choi et al., 2023 | 2023 | Cross sectional study | 181 | 63 ± 7 | 128 (70%) | - | - | - | 181 | 63 ± 6 | 131 (72%) | - |
| 44 | Moreno et al., 2023 | 2023 | Cross sectional study | 85 | 61 ± 14 | 35 (41%) | - | 12 ±13 | 49 ± 19.01 | 85 | 49±12 | 48(56%) | - |
| 45 | Gao et al., 2023 | 2023 | Cross sectional study | 58 | HGL: 57.44 ± 9.4  NGL: 59.71 ± 9.674 | 27 (46%) | HGL: 12.29 ± 3.64  NGL: 11.82 ± 3.23 | HGL: 11.83 ± 8.41  NGL: 7.82 ± 8.06 | HGL: 45.61 ± 12.61  NGL: 51.89 ± 10.19 | 42 | 54.19 ± 8.194 | 23 (54.7%) | 13.90 ± 4.11 |
| 46 | Zhang et al., 2023 | 2023 | Cross sectional study | 56 | 52.89 ±10.28 | 14 (25%) | 14.32 ± 2.24 | 7.37 ± 5.69 | 45.61 ± 11.74 | 60 | 54.35 ± 6.05 | 22(36%) | 13.81 ± 3.62 |
| 47 | Palix et al., 2022 | 2022 | Cross sectional study | - | - | - | - | - | - | - | - | - | - |
| 48 | Huang et al., 2022 | 2022 | Cross sectional study | 78 | 46.96 ± 9.56 | 49 (63%) | 11.33 ± 3.77 | 4.08 ± 3.58 | 42.88 ± 10.208 | 74 | 47.82 ± 10.31 | 48 (65%) | 10.98 ± 3.42 |
| 49 | Cui et al., 2023 | 2023 | Other: prospective study | 36 | 49.19 ± 5.879 | 13 (36%) | 11.00 ± 3.381 | 5.31± 5.21 | 43.88 ± 7.855 | 28 | 50.46 ± 6.374 | 10 (35%) | 10.86 ± 3.932 |
| 50 | Jing et al., 2023 | 2023 | Cross sectional study | 561 | 63.2 ± 6.9 | 54.4% | - | - | - | 569 | 59.8 ± 6.1 | 52.5% | - |
| 51 | Reynolds et al., 2023 | 2023 | Cross sectional study | 51 | 48.4 ± 11.3 | 38 (74.5%) | - | 20.1 ± 9.1 | 28.3 ± 14.5 | - | - | - | - |
| 52 | Chen et al., 2022 | 2022 | Cross sectional study | 68 | 47.71 ± 7.80 | 20 (29.4%) | 13.59 ± 2.94 | 7.03 ± 6.04 | 40.68 ± 9.86 | 68 | 45.76 ± 7.57 | 22(32%) | 13.96± 3.58 |
| 53 | Zhang et al., 2022 | 2022 | Cross sectional study | 81 | 54.15 ± 9.2 | 30  (37%) | - | 9.24 ± 6.61 | 44.91 ±11.32 | 48 | 54.13 ± 7.50 | 26(54%) | - |
| 54 | Lee et al., 2021 | 2021 | Cross sectional study | 30 | 54.2 ± 3.7 | 15 (50%) | - | 2.03 ± 1.62 | 52.17 ± 4.039 | 30 | 53.4 ± 5.1 | 15 (50%) | - |
| 55 | Monereo-Sánchez et al., 2023 | 2023 | Cross sectional study | 869 | 61.6 ± 7.9 | 313 (36%) | (l,m,h %) 41.2/28.9/29.9 | - | - | 3184 | 57.3 ± 8.5 | 1808 (56.8%) | (l,m,h %) 26.6/28.5/44.9 |

Table3. Studies Characteristics
