# Supplementary material for: A systematic review of the impact of type 2 diabetes on brain cortical thickness
Source: Front Dement. 2024 Jun 13;3:1418037. doi: 10.3389/frdem.2024.1418037 (PMC11285553; doi:10.3389/frdem.2024.1418037)
Supplement: Supplementary file 4 [file Table_4.DOCX]

|  | | | | | | | | | | | | |
| --- | --- | --- | --- | --- | --- | --- | --- | --- | --- | --- | --- | --- |
| **Ref.** | **MRI strength;** | **manufacturers** | **T1W Resolution info** | **Flip angle** | **Repetition time** | **Echo time** | **Scan time** | **Matrix size** | **Field of view (FOV)** | **Slice Thickness** | **Voxel size** | **Cortical thickness extraction software** |
| Schmidt et al., 2004 | 1.0- or 1.5-tesla |  | interslice gap of 1 or 1.2 mm | - | - | - | - | - | - | 5- or 6-mm thick | - | Not mentioned |
| Coutinho et al., 2017 | 3 T | Siemens Trio scanner (Siemens Medical Solutions, Erlangen, Germany) | T1-weighted whole-brain high-resolution (1—1—1 mm), and GRAPPA factor=2, | - | 2530 ms | 1.64, 3.50; 5.36 and 7.22 ms, | - | 256—256—176, bandwidth=651 Hz/pixel | 256—256 mm |  | - | FreeSurfer image suite version 5.3.0 |
|  |  |  |  |  |  |  |  |  |  |  |  |  |
| Buss et al., 2018 | 3T scanner (GE Healthcare, Ltd., UK) | using a 3D spoiled gradient-echo sequence | 162 axial-oriented slices for whole-brain coverage; 240-mm isotropic | 15 | 2.9 ms | 6.9 ms | 432 s. | - | 0.937-mm—0.937-mm | - | - | Freesurfer6.0 |
| Markus et al., 2017 | 1.5-T MRI system | (Magnetom Avanto, Siemens Medical Systems, Erlangen, Germany | - | 15° | 1900 msec | 3.37 msec | 3:38 min | - | - | - | 1.0x1.0x1.0 mm | FreeSurfer 5.1 |
|  |  |  |  |  |  |  |  |  |  |  |  |  |
| Garcia-Casares et al., 2014 | 3-Tesla whole-body research-dedicated scanner | (Intera, Philips Medical Systems,Best, the Netherlands) and an eight-channel head coil | - | 8 |  | 4.6 |  | 256/256 r | 240 mm | 1 mm (190 slices), | 0.9—0.9—1m | Statistical Parametric Mapping 8 software (SPM8) |
| (Roy et al., 2020 | a 3.0-Tesla MRI scanner | (Siemens, Magetom, Tim-Trio/Prisma, Erlangen, Germany) | inversion time = 900ms; | 9° | 2200ms | 2.34/2.41ms |  | 320—320; | 230—230 mm2 | 0.9mm |  | The statistical parametric mapping package (SPM12, MRIcroN, RESting-state fMRI data analysis Toolkit (REST) |
| Falvey et al., 2013 | 3T University of Pittsburgh. | Siemens TimTrio MR scanner at | (, imaging time [TI] = 900 ms, | 98 | 2,300 ms, | =3.43 ms |  | 256x224 | 256x224 mm2 | 176 slices, and 1 mm thick | 131 mm | using previously published methods |
| (Peng et al., 2014 | - | - | volume size 256h256h256, | - | - | - | - | - | - | - | 1h1h1 mm3 | The framework of the BrainLab toolbox |
| Last et al., 2007 | 5-mm slice thickness [1.5 mm skip] | - | TI=2,250 |  | 11,000 ms | 161 ms | - | 256x192 | 24x19 “cm2 | and 3-mm slice thickness | - | All image data were processed on a Linux workstation, using tools developed in the IDL programming environment (Re-search Systems, Boulder, CO) |
| Bernardes et al., 2018 | 3 Tesla | GE Signa HDxt scanner | - | - | - | - | - | - | - | 1 mm slice thickness |  | Freesurfer 5.3 and FSL-FIRST |
| Ferreira et al., 2017 | 3 T research scanner | (Magnetom TIM Trio, Siemens, Berlin,Germany) using a phased array 12-channel birdcage head coil(Siemens) | 176 single shot slices | 7° | - | - | - | - | 256 mm.x 256 mm | - | 1x1x1mm3 | MarsBaR toolbox, a Matlab toolbox forSPM which provides routines for the region of interest |
| Hayashi et al., 2011 | 1.5-T system | (Mahnetom Symphony, Siemens, Erlangen, Germany). | - | 158 | 11.4 | 4.4 | - | 256x256 | 22cm | 1.5mm | - | an MRI voxel-based specific regional analysis system developed for the study of Alzheimer’s disease (VSRAD) |
|  |  |  |  |  |  |  |  |  |  |  |  |  |
| Kumar et al., 2008 | 1.5 T scanner | - | - | - | - | - | - | - | - | - | - | automated software |
| Zhang et al., 2015 | Siemens 3Tesla TIM Trio MRI | system (Erlangen, Germany) | 176 slices | 9° | 1900 ms | 2.52 ms |  | 256 — 256 | 256 mm— 256 mm | 1 mm | 1 mm — 1 mm — 1 mm | Freesurfer 5.3.0 |
| Bruehl et al., 2009 | 1.5 T or 1.5 T | Siemens Avanto system and GE Vision | 192 slices | 15° | - | - | - | 256—128 | 250—250 mm2 | 1.2 mm |  | Not Mentioned |
| Gold et al., 2007 | - | - | T1-weighted spoiled gradient recalled sequence 124slices, no gap, | 60° | 9000 ms | 110 ms | 2 min 15 s | 256—128, | 250—250 mm2 | 1.5 mm |  | the locally developed Multimodal Image Data Analysis System (MIDAS) software |
| Korf et al., 2006 | 1.5-T | - | -- | - | - | - | - | - | - | - | - | Using MEDx version 3.41 software (Sensor Systems, Sterling, VA) |
| Wisse et al., 2014 | 1.5 Tesla (T) | Philips MRI scanner | recovery inversion time (TI): 410 ms) (FLAIR) /TI: 2000 ms)(both 38 contiguous 3voxels) and a sagittal 3D T1-weighted sequence (TI ms; 170) | - | 2919 ms | 22 ms | - | - | - | - | slices and 0.9 — 0.9 — 4.0 mm | Cerebral infarcts were manually segmented and added to the TBV. The hippocampus was manually segmented according to a previously described protocol |
|  |  |  |  |  | 6000 ms | 100 ms |  |  |  |  | 3voxels |  |
|  |  |  |  |  | 7.0 ms | 3.2 ms |  |  |  |  | slices;0.94 — 0.94 — 1.00 mm |  |
| Li et al., 2020 | T | Philips Achiev a scanner (Philips, the Netherlands) equipped with an 8-channel head coil | inversion time=900 ms | 8° | 9.8 ms | 4.6 ms | - | 256 — 256 | 256 — 256 mm | 1 mm |  | FreeSurfer 6.0.0 |
| Manschot et al., 2006 | 1.5 Tesla; | Philips MedicalSystems, Best, the Netherlands | TI: 2000, | - | 6000 | 100 | - | matrix 180256 | ,230 mm x 230 mm | slice thickness 4.0 mm |  | - |
|  |  |  | 38 slices |  |  |  |  |  |  |  |  |  |
| 35 | 1.5T | Philips magnetic resonance system | - | - | 234 ms | 2 ms | - | - | - | - | - | - |
| Ajilore et al., 2010 | 1.5-T | - | number of excitations=1.5; in-plane resolution=0.86—0.86 mm) | 45° | 20 ms | 6 ms; | - | 256—192 mm | 22 cm | thickness of 1.4 mm, no gaps | - | - |
| (Tchistiakova et al., 2014 | 3 T | Magnetom Trio Siemenssystemwith 12-channel head coil | TI = /1100 ms,, number of slices = 160 | 9° | 2000 ms | 2.63 ms | 6 min 30 s | 256 — 192 | 256 — 192 mm2 | 1 mm | - | Freesurfer 5.1.0 |
| Cui et al., 2014 | 3-Tesla | GE GHX MRI scanner using a quadrature and eight-channel phase array head coils (GEMedical Systems, Milwaukee, WI) | - | - | - | - | - | - | - | - | - | (IDL, Research Systems, Boulder, Colorado, USA) and MATLAB. Anatomical MR images (MP-RAGE and FLAIR) were co-registered non-linearly to the MNI152 standard template |
| Wennberg et al., 2016 | 1.5 Tesla | General Electric scanner using a standard multimodal protocol. | - | - |  |  |  |  |  |  |  | FreeSurfer 5.1 |
| Shaw et al., 2017 | 1.5 T | Siemens Avanto scanner | - | Wave1: 30° | Wave1: 28.05 ms | Wave1: 2.64 ms | - | Wave1: 256—256 | - | Wave 1: 2 mm |  | FreeSurfer v5.3 |
|  |  |  |  | Wave2: 8° | Wave2: 8.93 ms | Wave2: 3.57 ms |  | Wave2:256—256 mm |  | Wave 2: 1.5 mm |  |  |
|  |  |  |  | Wave3:1160 ms | Wave 3: 4.17 ms | Wave 3: 4.24 ms |  | Wave 3: 512—512 and |  |  |  |  |
|  |  |  |  | Wave3: 15° |  |  |  |  |  |  |  |  |
| Li et al., 2018 | 3-Tesla | Siemens Healthcare, Erlangen, Germany with a 12-channel phase-array head coil. | inversion time=900ms, 176 slices | 9° | 1900ms | 2.52ms | - | 256x256 | - | 1.0mm | 1mmx1mmx1mm | FreeSurfer software 5.3.0 |
| Shi et al., 2019 | 1.5T MRI | - | - | - | - | - | - | - | - | - | - | FreeSurfer v5.3 |
| Moran et al., 2019 | 3 Tesla | Siemens Magnetom TIM Trio with a phased array 12-channel birdcage head coil (Siemens, Munich, Germany | (TI)=1100ms; 176 single-shot interleaved slices with no gap with isotropic; | 7° | 2530 ms | 3.42 ms; |  |  | 256 mm x 256 mm |  | 111 mm | PM12(Wellcome Trust Centre for Neuroimaging, London, UK; andCAT12 toolbox (Structural Brain Mapping Group, JenaUniversity Hospital, Jena, Germany; |
| Crisóstomo et al., 2021 | 1.5 Tesla | MR unit (VISION MR, Siemens, Erlangen, Germany) | - | - | - | - | - | - | - | - | - | Not Mentioned |
| den Heijer et al., 2003 | 1.5 T | Philips MR system using a standardized protocol | (IR) (TR: 2919, TE: 22, (TI): 410), fluid attenuated inversion, TI: 2000) (all 38 contiguous slices) |  | 234 ms | 2 ms |  |  |  |  | 0.9 — 0.9 — 4.0 | Freesurfer software |
|  |  |  |  |  | recovery (FLAIR) (TR: 6000 | TE: 100 ms |  |  |  |  |  |  |
|  |  |  |  |  | a sagittal 3D |  |  |  |  |  | and a sagittal 3D |  |
|  |  |  |  |  | TR: 7.0 | a sagittal 3D |  |  |  |  | 0.94 — 0.94 — 1.00 |  |
|  |  |  |  |  |  | TE: 3.2 |  |  |  |  |  |  |
| Brundel et al., 2010 | 1.5 Tesla | (Signa Excite II, General Electric Healthcare, Milwaukee, WI, USA) | inversion time=400ms96 adjacent slices | 80 ° | 13.8ms | 2.8ms |  | 416—256 | 25cm x 25cm | 1.6 mm zero-padded to 0.8 mm |  | FreeSurfer |
|  |  |  |  |  |  |  |  |  |  |  |  |  |
| van Velsen et al., 2013 | a 3.0 Tesla | SIGNA EXCITE, GE Healthcare, Milwaukee, WI, USA), and a conventional eight-channel phased-array head coil | inversion recovery images: | 15° | 6.3 ms | 2.8 ms | - | 256 -256 | 24 cm24 cm | 4 mm | - | Free surfer (version 4.3.0) |
|  |  |  | TI = 2200 |  |  |  |  |  |  |  |  |  |
|  |  |  |  |  | 8802 ms | 124.3 ms, |  | 256-256 | 24 cm24 cm |  |  |  |
| Chen et al., 2015 | 1.5-Tesla MRI |  | - | - | - | - | - | - | - | - | - | FreeSurfer (version 5.3) |
| Moran et al., 2015 | a 3.0 Tesla | (SIGNAEXCITE, GE Healthcare, Milwaukee MR system, WI, USA), and a conventional eight- channel phased array head coil | 118 contiguous axial slices plane resolution:0.9375mm—0.9375mm, | 15 | 6.3 ms | 2.8ms | - | 256—256 | 24cm—24cm | - | - | newly developed BrainLab toolbox. |
| Peng et al., 2015 | 3.0 T | (SIGNA EXCITE, GE Healthcare, Milwaukee, WI, USA) | in-plane resolution:0.9375 mm— 0.9375 mm, | 15° | 6.3 ms | 2.8 ms | - | 256—256 | 24 cm—24 cm | - | - | Freesurfer software (version 4.3.0) |
| Chen et al., 2017 | 1.5-T | a standard 8-channel head coil (Siemens Avanto, Erlangen, Germany) | number of slices = 176, NEX = 1.0 | 15° | 1900 mm | 2.93 ms | 7 min 3 s | 128 — 128 | 240 mm — 240 mm | 1.0    mm | - | FreeSurfer image analysis software (vision 5.3.0) |
| Liu et al., 2019 | 3.0-Tesla | Siemens, MagnetomPrisma, Erlangen, Germany | inversion time = 900 ms | 9 ° | 2200 ms | 2.34/2.41 ms |  | 320—320 | 230—230 mm2 | 0.9 mm |  | is not mentioned |
| Choi et al., 2020 | 3.0-Tesla | Siemens 3.0-Tesla Trio Tim MRI scanner (Siemens AG, Erlangen, Germany) with a 12-channel phase-array head coil | inversion time (TI) = 1100 ms; number of slices = 176 | 9° | 1900 ms | 2.52 ms |  |  | 256 mmx256 mm | 1 mm | 1 mmx1 mmx1 mm | FreeSurfer software (version 5.3.0, Massachusetts GeneralHospital, Boston, MA, United States1). |
| Dong et al., 2022 | - | - | - | - | - | - | - | - | - | - | - | - |
| Honea et al., 2022 | - | - | - | - | - | - | - | - | - |  |  | MIMneuro Amyloid Workflow (version 6.8.7, MIM  Software Inc. |
| Choi et al., 2023 | 3.0-Tesla | Discovery MR750w, GE Healthcare,  Milwaukee, WI with a 24-channel head coil. | inversion time,  450 ms, number of slices, 154-172  according to head size | 12° | 8.5 ms | 3.2 ms | - | 256 x256 mm | 256 x256 mm2 | 1 mm | 1 mm | FSL-VBM,14 (http://fsl.fmrib.  ox.ac.uk/fsl/fslwiki/FSLVBM), an optimized  VBM protocol15 carried out with FSL tools. |
| Moreno et al., 2023 | 3.0-Tesla | Tim Trio scanner (Siemens,  Germany) equipped with a 12-channel birdcage head coil | inversion time  1100 ms, 176 slices | 7° | 2530 ms | 3.42 ms | - | - | 256—256 mm2 | - | 1—1—1 mm3 | FreeSurfer 7.0 |
| Gao et al., 2023 | 3.0-Tesla | 2-channel head coil  product on a MAGNETOM Skyra MR scanner (Siemens  Healthcare, Erlangen, Germany) | the number of slices=213 | - | 2300 ms | 2.32ms | - | - | 24cm—24cm | 0.9mm | - | statistical parametric mapping (SPM8) |
| Zhang et al., 2023 | 3.0-Tesla | (Ingenia, Philips Healthcare, The Netherlands) with a 16-  channel phased-array head coil. | the number of slices=328 | 8° | 7.5 ms | 3.5 ms | - | 256 x 256 | 250 mm—250 mm | 0.55 mm (no gap) | - | Computational Anatomy Toolbox 12 (CAT12: http://  www.neuro.uni-jena.de/cat/) software for SPM1 |
| Palix et al., 2022 | 3.0-Tesla | Discovery RX VCT 64 PET-CT device (General  Electric Healthcare | the number of slices=180 with no gap | 10° | 20 ms | 4.6 ms |  |  | 256 — 256 mm2 | 1 mm |  | Statistical Parametric Mapping version 12 software (SPM12) |
| Huang et al., 2022 | 3.0-Tesla | MAG-  NETOM Prisma, Siemens, Germany) equipped with a 64-channel head coil. | - | - | 2530 ms | 2.98 ms | 5 min 58 sec | - | 256 x 256 mm2 | 1 mm | 1—1—1 mm3 | FreeSurfer 7.2.0 |
| Cui et al., 2023 | 3.0-Tesla | Trio MRI system (GE  Discovery 750w 3.0T) equipped with a 32-channel phase-array head coil. | - | 12° | 8.7 ms | 3.2 ms | 4 min 23 sce | 256 — 256 | 256 x 256 mm2 | 1 mm | - | FMRIB Integrated Registration  and Segmentation Tool (FIRST) |
| Jing et al., 2023 | 3.0-Tesla | MRI scanner (Ingenia 3.0T; Philips, Best, the  Netherlands) | - | - | 6.7 ms | 3.0 ms | - | - | - | - | 1—1—1 mm3 | FreeSurfer software |
| Reynolds et al., 2023 | - | - | - | - | - | - | - | - | - | - | - | FreeSurfer software |
| Chen et al., 2022 | 3.0-Tesla | Discovery MR750w, General Electric, Milwaukee, WI, United  States) with a 24-channel head coil. | inversion  time (TI) = 450 ms, the number of slices=188 (sagittal slices) | 12° | 8.5 ms | 3.2 ms | 296 sec | 256 — 256 | 256 x 256 mm2 | 1 mm, no gap | - | the CAT12 toolbox, parametric mapping software (SPM122). Free Surfer |
| Zhang et al., 2022 | 3.0-Tesla | Siemens 3.0T Trio TIM MRI scanner  at the Henan Provincial Peoples Hospital | the number of slices= 160 | 9° | 1900 ms | 2.52 ms | - | 256 — 256 | 250 x 250 mm2 | 1 mm | 0.97— 0.97—1 mm | Statistical Parametric Mapping 12 (SPM12, https://www.fil.ion.ucl.ac.uk/spm/software/spm12/). |
| Lee et al., 2021 | 1.5-Tesla | whole-body  imaging system (Signa HDx, GE Healthcare, Milwaukee,  WI, USA) | - | 45° | 24 ms | 5 ms | - | 256 x 256 mm2 | 24 cm | 1.2 mm and no skip | - | FreeSurfer (Version 6.0) |
| Monereo-Sánchez et al., 2023 | 3.0-Tesla | MAGNETOM Prismafit, Siemens Healthineers GmbH) located at a  dedicated scanning facility (Scannexus, Maastricht, The Netherlands)  using a head/neck coil with 64 elements for parallel imaging. | the number of slices= 176 slices | - | 2300 ms | 2.98 ms | 900 ms | 256 — 240 | - | - | 1—1—1 mm3 | FreeSurfer (Version 6.0) |

**Table 4.** Imaging Characteristics
